# Utilization of the Inferior Epigastric Artery in Renal Transplantation for Patients With Severe Vascular Atherosclerosis: A Strategy to Optimize Graft Availability

**DOI:** 10.7759/cureus.77852

**Published:** 2025-01-22

**Authors:** Dionysios Prevezanos, Leonardos Chaikalis, Dimitrios Vlachos, Michail Mpelivanis, Christos Doudakmanis, Michail Konstantinidis, Ioannis Giannopoulos, Spiridon Vernadakis

**Affiliations:** 1 Propaedeutic Surgery, Athens Medical School, National and Kapodistrian University of Athens, Laiko General Hospital, Athens, GRC; 2 Renal Transplantation Unit, Laiko General Hospital, Athens, GRC; 3 Radiology, Laiko General Hospital, Athens, GRC

**Keywords:** complex transplantation, preoperative imaging, renal transplantation, surgical adaptability, vascular anastomosis

## Abstract

Renal transplantation is the preferred treatment for end-stage renal disease (ESRD); however, patients with significant vascular abnormalities may require innovative approaches to vascular anastomosis. This report describes a 72-year-old male with ESRD of unknown etiology who was assessed for a deceased donor kidney transplant. Severe atherosclerosis of the iliac arteries was identified on preoperative imaging, leading to the selection of the inferior epigastric artery (IEA) as the vascular conduit. Anastomosis was performed using interrupted 6-0 prolene sutures, resulting in successful graft reperfusion without intraoperative complications. Postoperatively, the patient experienced delayed graft function (DGF), remaining anuric for 10 days and requiring multiple hemodialysis sessions as well as antithymocyte globulin (ATG) therapy. Urine output resumed on the 10th postoperative day, and the patient was discharged two weeks after surgery. Despite initial challenges, including DGF requiring temporary dialysis, the patient achieved full recovery with stable graft function confirmed at follow-up. One-month follow-up with CT angiography confirmed satisfactory graft perfusion. This case underscores the feasibility of utilizing the IEA as an alternative vascular access in complex kidney transplantation, highlighting the critical importance of preoperative imaging, surgical adaptability, and the ongoing challenges of limited graft availability and long waiting times for transplant candidates.

## Introduction

Renal transplantation, first successfully performed in 1954, continues to be the gold standard and most cost-effective treatment for end-stage renal disease (ESRD) [1]. The procedure typically involves vascular anastomosis using the external iliac artery (EIA) due to its favorable anatomical location, appropriate caliber, and dependable blood flow. However, certain patient factors, such as vascular anomalies, extensive atherosclerosis, or a history of prior surgical interventions, can present significant challenges, necessitating alternative surgical approaches. Over the years, advanced imaging techniques like computed tomography (CT) have played a pivotal role in addressing these complexities. Notably, CT imaging has been reported to alter the surgical plan in approximately 25% of renal transplantation cases [2,3].

In situations where the anatomy is complex or there are comorbidities, one alternative approach involves utilizing the IEA for arterial anastomosis. Originally described by Dubernard et al. in 1976, the use of the IEA was initially employed for cases involving multiple renal vessels, demonstrating promising outcomes and broadening the range of surgical options for complex scenarios [4]. Although technically demanding, this method has proven to be a viable and effective solution in selected cases [5-10].

This report highlights the utilization of the IEA as an alternative conduit for arterial anastomosis in renal transplantation. By contributing to the growing body of evidence on innovative surgical strategies, we aim to highlight the potential of this technique to expand the repertoire of solutions available for challenging cases in renal transplantation.

## Case presentation

A 72-year-old male presented to our transplant center for evaluation as a candidate for kidney transplantation from a deceased donor. He was blood group O rhesus positive. The cause of his ESRD was undetermined, and he had been receiving maintenance hemodialysis since 2009, due to scarcity of renal grafts. His residual urine output was 50 mL, and his dry weight was 93 kg.

**Figure 1:**
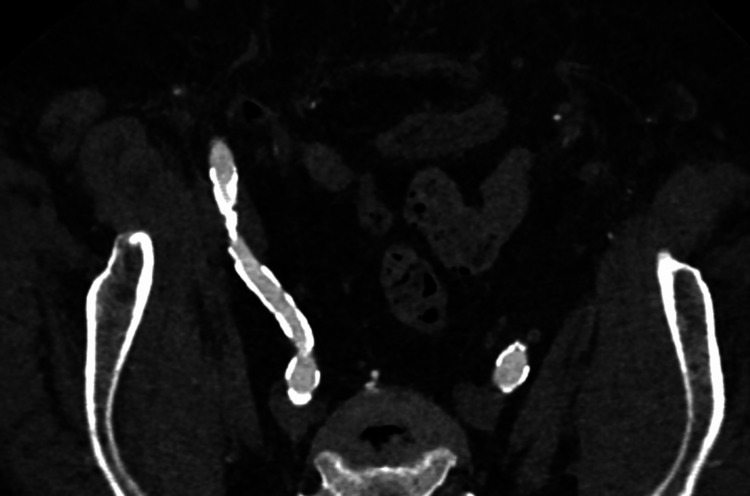
CT angiography demonstrating bilaterally heavily calcified EIA. CT, computed tomography; EIA, external iliac artery

**Figure 2:**
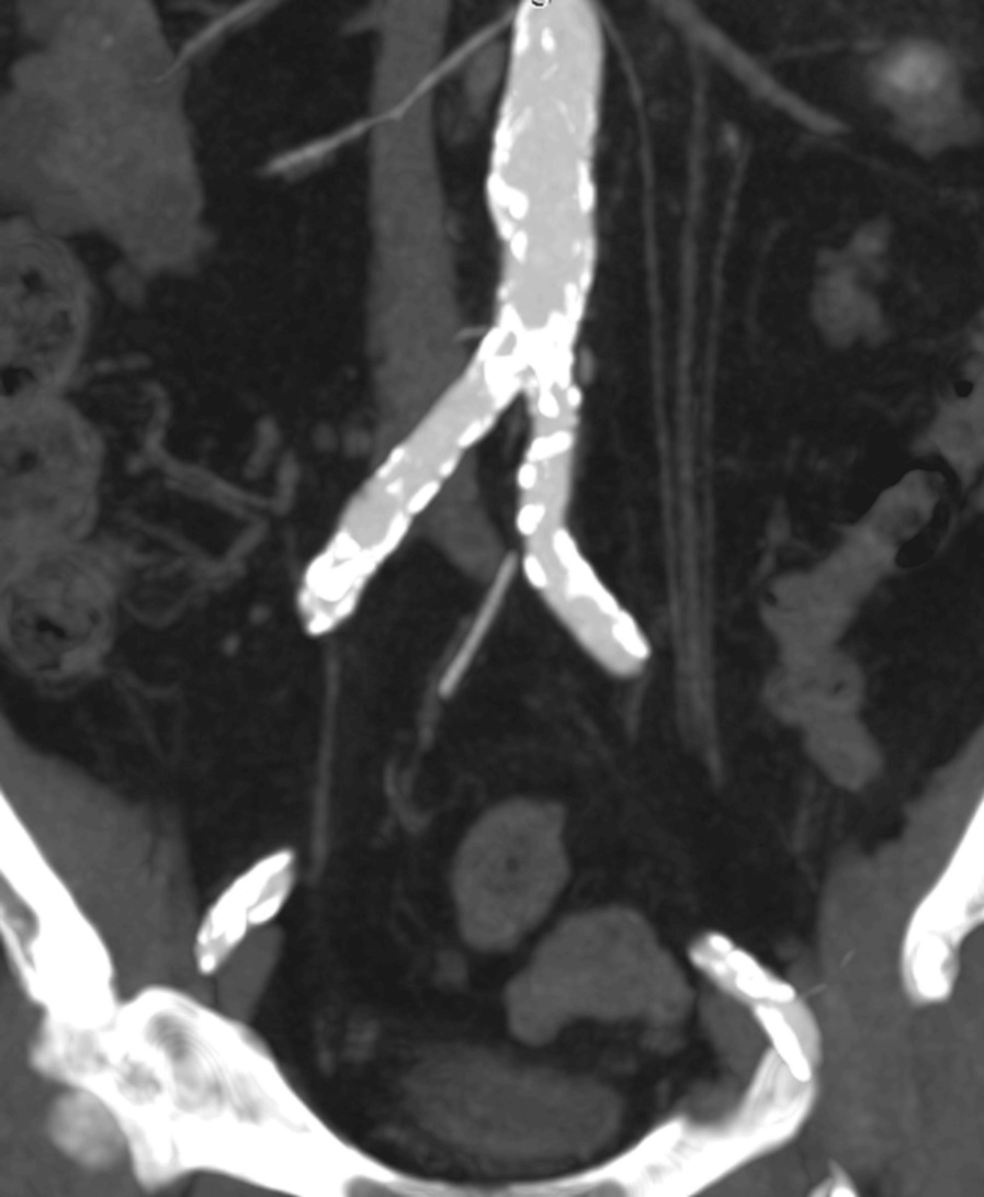
CT angiography demonstrating bilaterally heavily calcified CIAs. CT, computed tomography; CIAs, common iliac arteries

**Figure 3:**
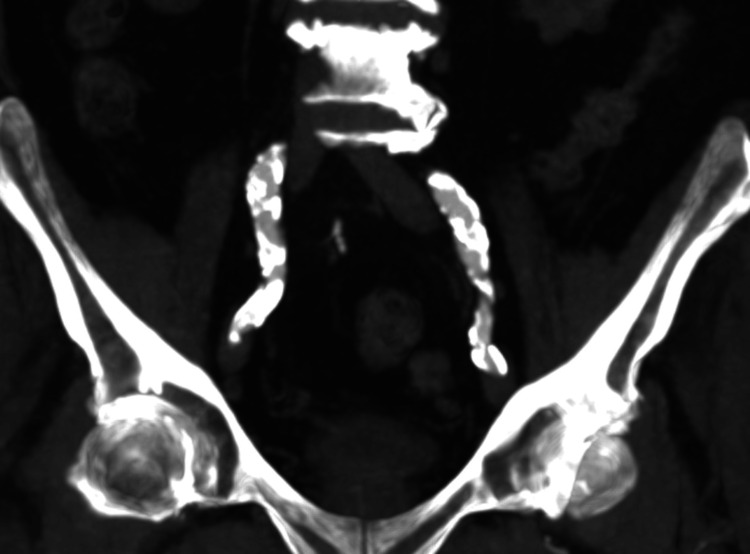
CT angiography demonstrating bilaterally heavily calcified EIAs. CT, computed tomography; EIAs, external iliac arteries

The patient’s medical history was notable for coronary artery disease, hepatic steatosis, gastritis, nodular goiter, arterial hypertension, and a previous open cholecystectomy performed via a midline incision. Pre-transplant imaging, including CT angiography, revealed significant atherosclerotic disease involving the iliac arteries, suggesting that the left EIA would be the most suitable site for graft placement (Figures 1-3). 

The donor was a 64-year-old female, classified as a donor after cardiac death (DCD) following a spontaneous cerebral hemorrhage. The patient remained at the Intensive Care Unit (ICU) for five days. CT demonstrated normal anatomy of the renal vessels. After completing all necessary tests to confirm brain death and obtaining written consent from the donor’s family, the organ procurement process was initiated. Her pre-donation renal function was assessed, with serum creatinine levels recorded at 1.2 mg/dL.

Intraoperatively, a left-sided hockey stick incision was utilized. The inferior epigastric vessels were identified and preserved due to concerns about the feasibility of an anastomosis with the EIA. Upon further dissection, the EIA was found to have severe atherosclerosis, making clamping and anastomosis with the renal artery of the graft unviable. The inferior epigastric artery (IEA), which had an appropriate diameter (about 5 mm), was chosen for the vascular anastomosis. The anastomosis was completed using interrupted 6-0 prolene sutures, and graft reperfusion was achieved without complications. The patient was transferred from the operating room in stable condition, without the need for intraoperative blood transfusion, but remained anuric postoperatively.

The graft’s cold ischemia time was 15 hours and 30 minutes, and the warm ischemia time was 40 minutes. The induction therapy included basiliximab and the antibiotic regimen was given based on the deceased donor's antibiogram. During surgery, the patient was administered 500 mg of Solu-Medrol and 20 ampules of Lasix.

On postoperative day 1, a diethylenetriamine pentaacetate (DTPA) renal scan demonstrated reduced perfusion and impaired excretion of the radiotracer. The patient initially received a triple immunosuppressive regimen consisting of tacrolimus, mycophenolic acid, and prednisolone. Tacrolimus dosing was adjusted based on serum levels, while Mycophenolic Acid was tailored to WBC counts to prevent cytopenia. The patient remained anuric for 10 days, during which he required five hemodialysis sessions and received antithymocyte globulin empirically (ATG). Several factors could have contributed to the delayed graft function (DGF), including the donor’s advanced age, prolonged cold ischemia time, the recipient's age, and comorbidities, as well as the impact of the long-term waiting list, which may have led to recipient deterioration prior to transplantation. On the 10th postoperative day, the patient began to produce urine and the patient's creatinine was 2.5 mg/dL. He was discharged two weeks after the transplantation with a creatinine level of 2.1 and producing 1.5 liters of urine.

**Figure 4:**
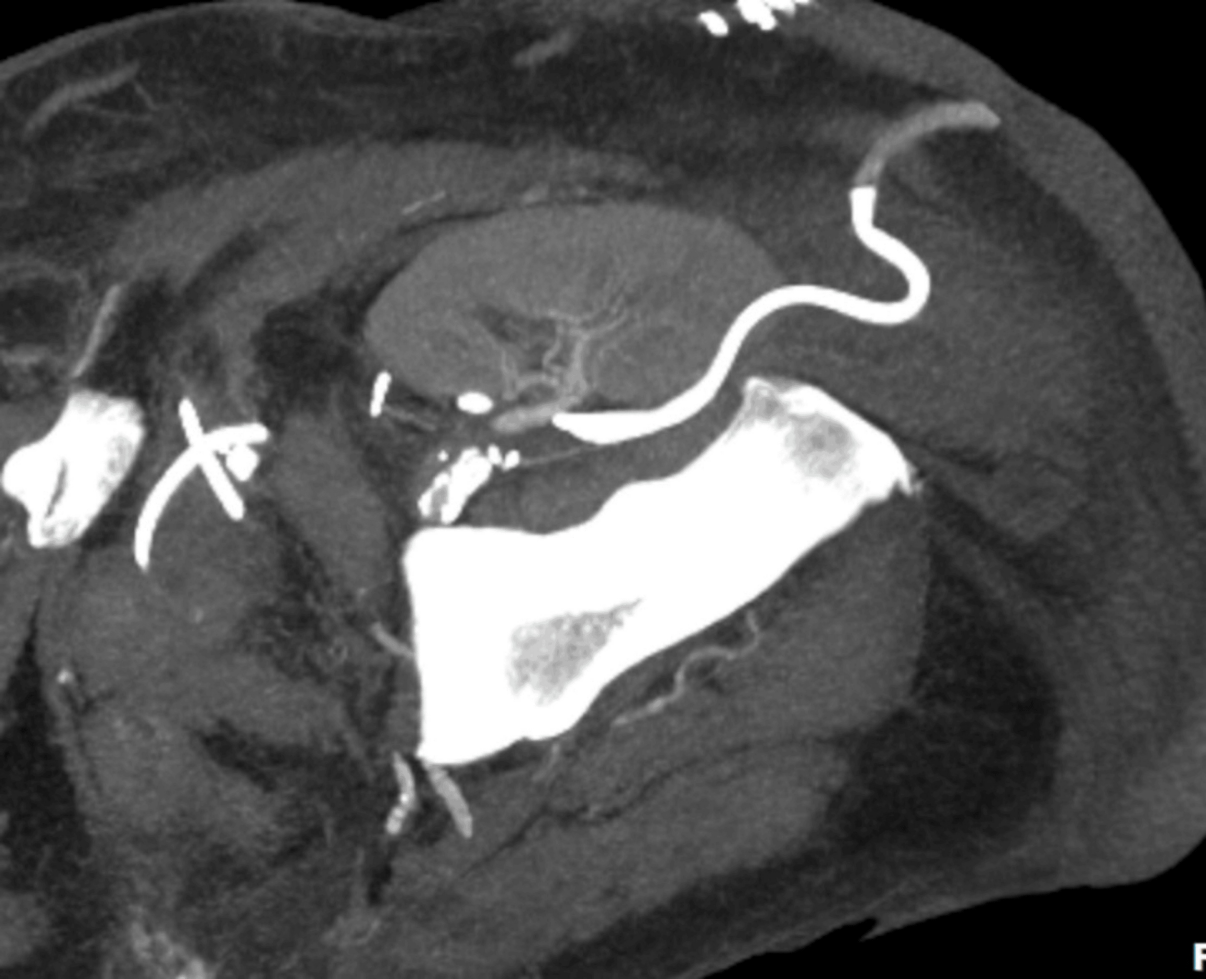
CT angiography demonstrating adequate perfusion of the renal graft. CT, computed tomography

At a one-month follow-up, a repeat CT angiography was performed due to stable creatinine levels. The CT angiography confirmed adequate perfusion of the transplanted kidney (Figure 4). The patient subsequently underwent follow-up with the Nephrology department, but further records of his care are unavailable.

## Discussion

The IEA serves multiple purposes in surgical practice, being widely utilized for various reconstructions, including coronary artery bypass grafting [11]. Its first documented use in renal transplantation dates back to 1976 [4]. Since then, the presence of multiple renal arteries has frequently prompted surgeons to adopt this technique [5-10], achieving satisfactory graft perfusion in these scenarios.

In one instance, the IEA was successfully employed in a case involving the splitting of a cadaveric horseshoe kidney to address the shortage of renal grafts, ensuring adequate perfusion [10]. Additionally, Giuseppe Serena et al. described the use of the IEA as an alternative to extending a short upper pole artery. This was accomplished through an end-to-side anastomosis between the upper pole arterial branch and the main renal artery using 8-0 prolene sutures [12].

Beyond the IEA, other alternative conduits have also been explored. For example, a reversed ovarian vein was successfully utilized as a vascular prosthesis [13].

It is important to emphasize the role of imaging modalities, particularly CT angiography, in anticipating and addressing such challenges. These imaging techniques are invaluable in identifying complications such as multiple renal arteries and extensive atherosclerosis, allowing for better preoperative planning and prevention of adverse outcomes [2,3,14,15].

Similar to previously reported cases, where the IEA was successfully utilized for graft perfusion in complex scenarios, our case demonstrates its efficacy in achieving adequate perfusion and graft functionality. However, unlike other instances where the IEA was used for anatomical anomalies or multiple renal vessels, our case highlights the use of this vessel to circumvent severe atherosclerosis, emphasizing the critical role of preoperative imaging in identifying and addressing vascular challenges. This approach aligns with established practices while reinforcing the adaptability of the IEA in unique transplant scenarios.

## Conclusions

This case highlights the successful use of the deep IEA as an alternative vascular conduit for arterial anastomosis in renal transplantation. In a patient with extensive atherosclerosis of the EIA, this technically challenging approach (pre-operative planning, severe recipient's atherosclerosis, and small caliber IEA) provided a viable solution, ensuring adequate graft perfusion and functionality. Postoperative recovery was initially complicated by DGF requiring temporary hemodialysis support, but the patient ultimately achieved satisfactory urinary output and graft perfusion, as confirmed by follow-up imaging. This case underscores the importance of surgical adaptability and the potential utility of alternative vascular techniques in complex renal transplantation scenarios.
